# Impact of Cumulative Corticosteroid Dosage on Preventable Hospitalization among Taiwanese Patients with Ankylosing Spondylitis and Inflammatory Bowel Disease

**DOI:** 10.3390/jcm8050614

**Published:** 2019-05-07

**Authors:** Beth I. Wallace, Chelsea A. Harris, Lu Wang, Mochuan Liu, Jung-Sheng Chen, Chang-Fu Kuo, Kevin C. Chung

**Affiliations:** 1 Center for Clinical Management Research, VA Ann Arbor Healthcare System, Ann Arbor, MI 48105, USA; brennerb@med.umich.edu; 2 Division of Rheumatology, Department of Internal Medicine, University of Michigan, Ann Arbor, MI 48109, USA; 3 Section of Plastic Surgery, University of Michigan, Ann Arbor, MI 48109, USA; Chelsea.a.harris@gmail.com; 4 Department of Biostatistics, School of Public Health, University of Michigan, Ann Arbor, MI 48109, USA; luwang@umich.edu (L.W.); mochuan@umich.edu (M.L.); 5 Division of Rheumatology, Allergy and Immunology, Chang Gung Memorial Hospital, Taoyuan 33305, Taiwan; rschen0404@gmail.com; 6 Division of Rheumatology, Orthopaedics, and Dermatology, School of Medicine, University of Nottingham, Nottingham NG7 2RD, UK

**Keywords:** autoimmune disease, preventable hospitalization, corticosteroid

## Abstract

**Background:** Corticosteroids are commonly prescribed for autoimmune conditions, but their impact on preventable hospitalization rates is unclear. This study sought to investigate the effect of corticosteroid use on hospitalization for ambulatory care sensitive conditions among Taiwanese patients with ankylosing spondylitis (AS) or inflammatory bowel disease (IBD). **Methods:** This was a retrospective cohort study using adults in the Taiwan National Health Insurance Research database receiving a new diagnosis of AS (*n* = 40,747) or IBD (*n* = 4290) between January 2002 and June 2013. Our main outcome measure was odds of preventable hospitalization for eight ambulatory care-sensitive conditions defined by the Agency for Healthcare Research and Quality. **Results:** In the first quarter (three months) following diagnosis, corticosteroid usage was common among patients with AS and IBD (18.5% and 30%, respectively). For every 100 mg increase in corticosteroid dose per quarter, adjusted odds of preventable hospitalization in the following quarter increased by 5.5% for patients with AS (aOR = 1.055, 95% CI 1.037–1.074) and 6.4% for those with IBD (aOR = 1.064, 95% CI 1.046–1.082). **Conclusions:** Relatively low doses of corticosteroids significantly increase AS and IBD patients’ short-term odds of hospitalization for ambulatory care-sensitive conditions. As recommended by current clinical guidelines, physicians should use corticosteroids sparingly in these populations, and prioritize initiation/escalation of disease-modifying anti-rheumatic drugs for long-term management. If corticosteroids cannot be avoided, patients may require monitoring and/or prophylaxis for corticosteroid-associated comorbidities (e.g., diabetes) which can result in preventable hospitalizations.

## 1. Introduction

Our global healthcare environment has become increasingly focused on maximizing value. This is of particular importance when managing high-cost, low-prevalence conditions such as autoimmune diseases, for which expensive biologic disease-modifying anti-rheumatic drug (biologic DMARD) treatments are often required for management of more severe patients. When treating such conditions, it is imperative to weigh the net benefits of biologic DMARDs against cheaper, more readily available options such as corticosteroids, which have long been used for symptom relief of autoimmune disease, and more recently shown to play a disease-modifying role in certain autoimmune conditions [1,2,3]. 

To date, the overall value of low-dose corticosteroid medication for autoimmune disease management has been notoriously difficult to define [1,2,4]. Systemic corticosteroids are more efficacious in some autoimmune conditions than they are in others [1,4,5], and have well-studied side effects including myocardial infarction, hypertension, congestive heart failure, infection, and death [6,7,8,9,10,11], which depend on both the amount of corticosteroid exposure and the risk factors of the treated patient [12]. As a result, clinical guidelines for many autoimmune conditions cannot provide specific corticosteroid dosing recommendations [1,2,4,13]. Additionally, there have been limited studies of corticosteroid safety and effectiveness in patients of Asian descent, despite previous work showing high prevalence of autoimmune conditions [14] and unique adverse DMARD side effect profiles [15,16] in such populations. It is; thus, important to understand how corticosteroids impact the risk-benefit relationship between treating autoimmune disease and predisposing patients to the corticosteroid-related adverse events discussed above, particularly in Asian populations.

We sought to use the Taiwan National Health Insurance (NHI) database to investigate how oral corticosteroid utilization for adult patients with ankylosing spondylitis (AS) and inflammatory bowel disease (IBD) affects hospitalization rates for eight ambulatory care-sensitive conditions, for which effective outpatient care may reduce the need for hospitalization. As Taiwan provides universal health coverage and free prescription medications, this dataset offers an unparalleled opportunity to quantify corticosteroids impact in an Asian population specifically.

## 2. Patients and Methods

### 2.1. Participants: Defining the Cohort and Baseline Health Status

The Taiwan National Health Insurance (NHI) database includes 28.75 million living and deceased Taiwanese beneficiaries (over 99% of the population) and contains both registration files and original claims [17]. This study was approved by the Institutional Review Board of Chang Gung Memorial Hospital (201700359B1) as well as the data holder of the NHI database.

We used validated claims definitions for IBD [18] and AS [19] to identify Taiwanese patients enrolled in NHI between January 2002 and June 2013 who received a new diagnosis of either condition while enrolled (Supplemental Figure S1) (Supplemental Table S1). We chose AS and IBD because systemic corticosteroids have no role in managing AS apart from symptom relief [4], but are a guideline-based part of short-term IBD management [4,20]. We defined index date (*t* = 0) as a patient’s first captured AS or IBD medical claim after a one-year enrollment period without medical claims for AS or IBD or pharmacy claims for DMARDs (“washout period”) prior to the index date (Supplemental Table S2). As we had access to complete data through December 2013, the study period was truncated in June 2013 to ensure all included subjects had at least six months of post-diagnosis data. We excluded patients with incomplete demographic data for age or gender, age younger than 18 years at index date, or diagnosis codes for both AS and IBD. 

Information obtained for all included patients included demographic data, measures of baseline health status and healthcare utilization, and measures of autoimmune disease-associated healthcare utilization. Demographic data obtained included age, sex, occupation, urbanization, and income quartile. Data on baseline health status included baseline Elixhauser comorbidity index [21,22] calculated during the washout period, as well as a modified version of the Elixhauser index which excluded eight corticosteroid-sensitive comorbidities (diabetes, hypertension, congestive heart failure, rheumatoid arthritis, peripheral vascular disease, obesity, and peptic ulcer disease). Data on baseline healthcare utilization included counts of outpatient physician visits and inpatient admissions during the washout period. Data on autoimmune disease-associated healthcare utilization included number of visits to a rheumatologist (for AS) or gastroenterologist (for IBD) within six months after index date, and use and dose of DMARD medications (Supplemental Table S2). We included hydroxychloroquine because patients with AS and IBD often have nonspecific musculoskeletal and gastrointestinal presentations which overlap with other autoimmune diseases [23,24]. For this reason, we did not exclude other autoimmune diseases in defining our cohort, such that some patients may be prescribed hydroxychloroquine for coexisting conditions, such as rheumatoid arthritis or lupus, or for features of their AS or IBD (i.e., oral ulcers, peripheral arthritis, rash) which mimic such conditions. As hydroxychloroquine is an immunomodulator which also impacts both blood sugar and blood pressure [25], it also seemed appropriate to control for its use in evaluating our outcome. 

### 2.2. Medication Dosage

We captured all outpatient pharmacy claims for synthetic and biologic DMARD medications (Supplemental Table S2) and oral corticosteroid medications (Supplemental Table S3) occurring during the study period, which extended from 90 days pre-index date (day –90) through 365 days post-index date (day 365) (Supplemental Figure S2). We chose this period as encompassing the time immediately before AS or IBD diagnosis when a patient might begin to seek care for associated symptoms, as well as the early post-diagnostic period when DMARDs may not yet have been prescribed, or symptoms may be inadequately treated by newly initiated/escalated DMARD regimens. This study period was then split into three-month calendar quarters, with quarter –1 (Q-1) representing days –90 to –1, quarter 1 (Q1) representing days 0 to 91, quarter 2 (Q2) representing days 92 to 183, quarter 3 (Q3) representing days 184 to 275, and quarter 4 (Q4) representing days 276 to 365. 

We calculated cumulative doses of oral corticosteroid, synthetic DMARD, and biologic DMARD medications, and oral corticosteroid days’ supply, dispensed in each calendar quarter during the study period. Corticosteroid dose was converted to mg prednisone equivalents (Supplemental Table S3). Each DMARD medication dose was scaled to the median quarterly dose (in mg) observed among users of that medication in NHI database with AS and IBD, respectively (Supplemental Table S4). We addressed medication overlaps differently, depending on drug class. Based on a senior author’s (CFK) clinical expertise and the fact that the Taiwanese government fully subsidizes medication costs, we assumed that, in cases of overlapping corticosteroid prescriptions, patients switched to the new corticosteroid regimen immediately. We made the same assumption for identical synthetic DMARDs and overlapping biologic DMARDs. In contrast, patients with AS and IBD often take two synthetic DMARDs or a synthetic and a biologic DMARD concurrently [1,4,20]; thus, we counted the full dosage for each DMARD prescription dispensed in these cases (Supplemental Figure S3). Finally, because granular inpatient drug dose and duration data were not available in the NHI database, inpatient medications were not considered in our analysis.

### 2.3. Outcome: Rate of Preventable Hospitalization for Ambulatory Care-Sensitive Conditions

Our primary outcome measure was hospitalization with a primary diagnosis of eight ambulatory care-sensitive conditions previously defined by the Agency for Healthcare Research and Quality (AHRQ). Ambulatory care-sensitive conditions are medical conditions for which effective outpatient care may reduce the need for hospitalization among adults (“preventable hospitalizations”) (Supplemental Table S5) [26]. We selected the following eight ambulatory care-sensitive conditions from the sixteen defined by AHRQ: uncontrolled diabetes, short-term or long-term complications of diabetes, lower extremity amputation among patients with diabetes, hypertension, congestive heart failure, bacterial pneumonia, or urinary tract infection. We selected these conditions because they are (a) known complications of corticosteroid use [6,7,8,9,10,11]; and (b) not conditions for which corticosteroids are an established part of routine disease management, as in asthma or chronic obstructive pulmonary disease. 

### 2.4. Statistical Analysis

To quantify the impact of corticosteroid usage on risk of future preventable hospitalizations in the AS and IBD cohorts, we independently fitted a generalized linear mixed effect models for each cohort. Our primary outcome was longitudinal preventable hospitalization with a primary diagnosis of any of the eight ambulatory care-sensitive conditions above (Supplemental Table S5), assessed during each three-month calendar quarter of the study period. Our primary exposure was cumulative corticosteroid dosage in the preceding three-month calendar quarter. We scaled our analysis to detect the effect of a 100 mg increase in cumulative corticosteroid dose, which represents the effect of a single short burst of high-dose corticosteroids (20 mg prednisone or equivalent given for five days). We applied a random intercept logistic regression model adjusting for demographic factors, baseline health and healthcare utilization, and disease-associated healthcare utilization, as well as an indicator variable designating presence of previous preventable hospitalization during the current quarter. The random intercept was included in the model to accommodate the within-subject correlations, and covariates are selected by its significance in the model vis backward model selection procedure. The random effect models allow unbalanced data and missing at random, which is a reasonable assumption in our study. The final covariates included in our model are listed in Supplemental Table S6. All statistical analyses were conducted using standard functions in SAS 9.4. 

## 3. Results

### 3.1. Cohort Demographic Data

We analyzed an AS cohort of 40,747 patients, and an IBD cohort of 4290 patients (Figure S1). Baseline demographic data, baseline health status and healthcare utilization statistics, and a summary of preventable hospitalizations during follow-up are presented in Table 1. Compared to patients with IBD, AS subjects were younger on average, and higher percentages saw specialists within the first six months after diagnosis and worked in white-collar jobs. IBD patients had higher unadjusted mean baseline Elixhauser comorbidity scores than AS patients (1.09 versus 0.50). Unadjusted rate of preventable hospitalization was 2.89% in the AS group and 12.84% in the IBD group. The most common examined causes of preventable hospitalization during the study period were bacterial pneumonia (1.38% in AS group, 6.36% in IBD group) urinary tract infection (1.06% in AS group, 5.66% in IBD group) and long-term complications of diabetes (0.43% in AS group, 2.10% in IBD group).

### 3.2. Corticosteroid and DMARD Usage

Corticosteroid exposure by calendar quarter, beginning three months prior to index date and ending 12 months after the index date, is presented in Table 2. Corticosteroid and DMARD utilization over the study period is presented in Table 3. Utilization of oral corticosteroids among IBD patients in this cohort was similar to that previously reported in American claims analyses [27]. There are few published reports of systemic corticosteroid use in AS, as its use is not a recommended part of AS management [4]. Median daily doses were similar between the two groups, and remained ≤10 mg/day for all quarters evaluated. 

In the AS group, peak corticosteroid usage occurred in the three months after AS diagnosis (Q1), when 18.5% of patients received corticosteroids at a median daily dose of 7.9 mg/day (mean 9.7 mg/day, IQR (4.6, 13.0)) for a median duration of 16 days (mean 28.4 days, IQR (6, 44)). By Q4, only 11.3% of AS patients received corticosteroids, at median daily dose 6.7 mg/day (mean 8.9 mg/day, IQR (3.1, 12.0)). In the IBD group, corticosteroid utilization remained close to 30% across the entire year post-diagnosis, and duration of corticosteroid exposure per quarter increased with time (median (IQR) 39 (10, 74) days in Q1, and 51 (15, 85) days in Q4). Median daily corticosteroid dose in IBD cohort was stable and similar to that of the AS cohort during this period, ranging from 8.0 mg/day (mean 9.6 mg/day, IQR (5.0, 13.7)) in quarter 1 to 7.8 mg/day (mean 9.3 mg/day IQR (5.0, 12)) in Q4.

### 3.3. Cumulative Corticosteroid Usage and Preventable Hospitalization

**Figure 1:**
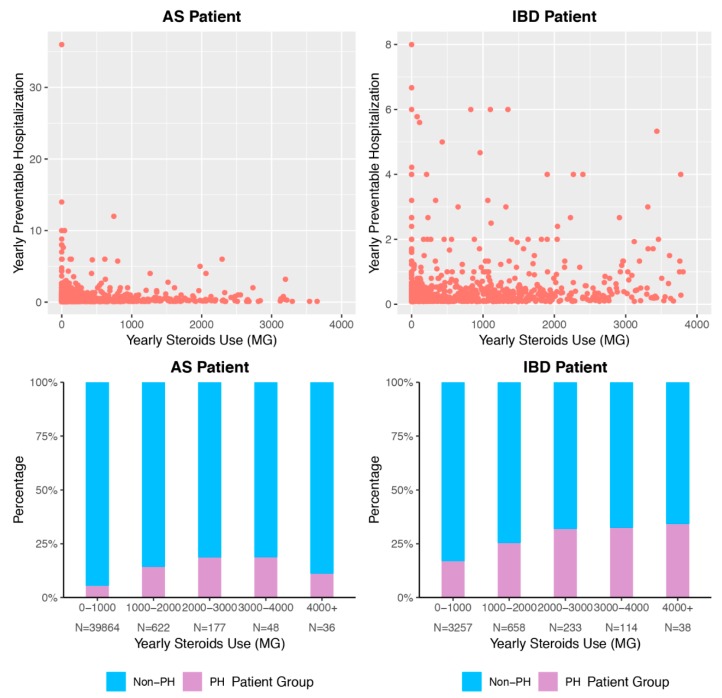
Association of Cumulative Steroid Exposure and Preventable Hospitalization. Density of points describes marginal association between cumulative corticosteroid dose and cumulative hospitalizations for ambulatory care-sensitive conditions. Bar graphs indicate percentage of patients per corticosteroid dose range who experience at least one hospitalization for ambulatory care-sensitive conditions during the study period. PH = preventable hospitalization, meaning hospitalization for ambulatory care-sensitive condition. AS = ankylosing spondylitis. IBD = inflammatory bowel disease.

We explored the unadjusted relationship between cumulative yearly corticosteroid dose and number of preventable hospitalizations over the study period (Figure 1). A preliminary analysis shows that higher cumulative corticosteroid exposure seems, in general, to be associated with more hospitalizations. However, it is important to note that across all dosage categories, the majority of both AS and IBD patients had no preventable hospitalizations. 

### 3.4. Association Between Corticosteroid Usage and Preventable Hospitalization

After controlling for patient demographic data, baseline health status and healthcare utilization, frequency of specialist visits, and DMARD usage, increased quarterly corticosteroid dosage in a given calendar quarter was significantly associated with increased odds of preventable hospitalizations in the following quarter, in both the AS and IBD cohorts (Table 4). A 100 mg increase in cumulative quarterly corticosteroid dose increased adjusted odds of preventable hospitalization by 5.5% among AS patients (adjusted odds ratio (OR) = 1.055, 95% CI (1.037, 1.074), *p* < 0.0001) and 6.4% among IBD patients (adjusted OR = 1.064, 95% CI (1.046, 1.082), *p* < 0.0001). A sensitivity analysis replacing the standard Elixhauser comorbidity index Score with a modified score excluding corticosteroid-sensitive conditions did not change this effect substantially (Supplemental Table S7). In the IBD population, no synthetic or biologic DMARDs were significantly associated with a change in odds of preventable hospitalization in a model which also included corticosteroid use. In the AS population, only hydroxychloroquine and etanercept were associated with increased hospitalizations (adjusted OR and 95% CI of 1.241 (1.015–1.518), *p* = 0.035 and 1.790 (1.009–3.177), *p* = 0.047, respectively). 

## 4. Discussion

In a national longitudinal sample of Taiwanese patients with AS and IBD, exposure to a single short-term burst of oral corticosteroids (100 mg prednisone or equivalent), during a three-month calendar quarter, independently increased the risk of hospitalization for eight corticosteroid-associated ambulatory care-sensitive conditions by 5%–6% over the subsequent calendar quarter. Furthermore, corticosteroid use was found to be common among Taiwanese patients with both AS and IBD, with 30% of IBD patients continuing to receive 50 days or more of moderate dose corticosteroids (8–9 mg/day prednisone equivalent) up to a year after their diagnosis, with duration of exposure increasing over time. These findings occur despite a lack of evidence that corticosteroids have net benefit for management of AS [4], and despite recommendations that corticosteroids be used only for short-term IBD management [4,20].

This study is among the first to evaluate how corticosteroids affect likelihood of hospitalization for ambulatory care-sensitive conditions among patients with autoimmune disease, and provides previously unavailable longitudinal information about how corticosteroids are used to manage AS and IBD in Taiwan. Our work is supported by a previous registry cohort study in rheumatoid arthritis [28] that demonstrated that corticosteroids increase hospitalization risk for pneumonia. However, our work is the first to show this effect in the Taiwanese population, uses a more generalizable AS/IBD national cohort, and considers a broader range of conditions. 

Our work, though consistent with existing guidelines for AS and IBD management [4,20], goes further in suggesting that even short-term use of corticosteroids may be associated with avoidable harms in both populations. As a result, increased use of DMARD therapy, although likely costlier in the short-term than use of corticosteroids as monotherapy, may potentially result in net cost savings if its use is able to prevent corticosteroid-associated preventable hospitalizations while also maintaining autoimmune disease control. Further work, beyond the scope of this manuscript, is required to evaluate this hypothesis. Additionally, though monitoring for complications, such as hypertension or elevated blood glucose, is recommended for patients receiving moderate-to-high dose corticosteroids [29], these recommendations are vague, cannot account for patient-level variation, and do not provide recommendations for patients receiving short-term or low-dose corticosteroids. Thus, in cases where even short-term corticosteroids are clearly indicated (i.e., acute IBD flare), prescribing clinicians, as well as primary care physicians, should consider escalating monitoring for these conditions, which otherwise might go unreported by the patient until severe. At the local level, precisely tracking rates of preventable hospitalization for ambulatory care-sensitive conditions (as proposed by AHRQ for all Prevention Quality Indicators) may help identify which corticosteroid-associated disease processes would respond best to increased preventive care. 

Our study has several limitations. Our use of claims data prevented direct assessment of AS and IBD disease severity and resulting confounding by indication. However, the ambulatory care-sensitive conditions we chose to evaluate are clinically unlikely to be the direct result of inadequately controlled IBD or AS; in other words, it does not seem clinically likely that having more severe AS or IBD of itself places a patient at higher risk of hospitalization for the specific conditions examined here. It is quite plausible that patients with more severe AS or IBD are more prone to such hospitalizations by virtue of higher corticosteroid and/or DMARD exposure, and their risk of hospitalization may also differ based on baseline health status or level of medical contact. We thus control for DMARD use, baseline health status, and medical contact in our model. We were also unable to account for inpatient drug use in our model due to lack of granular data on inpatient drug dose and duration. As inpatient use of DMARD to treat IBD and AS is very uncommon, this primarily impacts our estimation of corticosteroid exposure. Since including inpatient corticosteroid exposure in our model would be likely to result in higher estimates of hazard (i.e., excluding inpatient corticosteroid exposure biases our results to the null), it seems unlikely that including it would change our conclusions.

Because we used Taiwanese data, differences in care utilization patterns specific to Taiwan or to countries with single-payer health systems (i.e., lower use of DMARDs, increased frequency of specialist visits) may limit the generalizability of our results. Furthermore, low rates of DMARD utilization prevented a descriptive analysis of DMARD utilization patterns. This is in part owing to the fact that biologic DMARD were not widely available for IBD management in Taiwan until after 2013, when our data analysis ends; however, biologic DMARD were available for AS management during our study period, and rates of use were not substantially different between the two cohorts. It is; therefore, not clear whether the persistent high corticosteroid use seen in the IBD population was owing to inadequate DMARD escalation or inappropriate continued use of corticosteroids once effective DMARD treatment was established. It is also notable that 58% of our IBD cohort received hydroxychloroquine, which is not typically a part of luminal IBD treatment, and are seen fairly frequently by rheumatologists. As IBD is an uncommon diagnosis in Taiwan, and often has features overlapping with other autoimmune diseases (i.e., oral ulcers, enteric arthritis), we suspect that many of our IBD patients were treated with hydroxychloroquine by their rheumatologist for coexisting autoimmune diseases like rheumatoid arthritis or lupus, or IBD manifestations mimicking such conditions (i.e., enteric arthritis, oral ulcers, rash, etc.). Further work is warranted to evaluate practice patterns of IBD management in Taiwan, as these are not well characterized.

Our analysis also did not examine to what degree increases in preventable hospitalizations associated with corticosteroids were balanced by decreases in IBD-related hospitalizations. As current IBD management guidelines recommend against long-term corticosteroid use [1,20], it is reasonable to assume that the degree of reliance on corticosteroids seen in our analysis, and any resulting increase in preventable hospitalizations related to ambulatory care-sensitive conditions, is likely inappropriate regardless. In AS, where systemic corticosteroids are not thought to be effective management, it is quite unlikely that use of steroids would reduce hospitalizations due to AS activity. Although we adjusted for overall comorbidity, we did not adjust for specific preexisting comorbidities (e.g., preexisting diabetes), which might predispose to our selected outcome once patients were exposed to corticosteroids. However, the effects on hospitalization were seen with extremely low corticosteroid doses, and use of a modified Elixhauser score did not change these effects. Finally, our analysis may suffer from lead time bias, in that patients who received high corticosteroid doses prior to cohort entry may not have been followed for long enough to fully manifest complications, particularly if they are young. However, our decision to restrict our cohort to incident IBD/AS patients would tend to bias toward the null.

In a national Taiwanese sample, patients with incident AS and IBD use oral corticosteroids commonly, with a dose of corticosteroid equal to that commonly given in a single short-term burst associated with a significantly increased risk of hospitalization for eight corticosteroid-associated ambulatory-care sensitive conditions. Before prescribing corticosteroids, providers should carefully consider the risks and benefits such treatment will pose for each patient, and even short-term corticosteroid use should prompt consideration of close monitoring for known corticosteroid-associated side effects. 

## Figures and Tables

**Table 1:** Study subject characteristics.

Variable	AS Group *n* = 40,747	IBD Group *n* = 4290
**DEMOGRAPHICS**		
**Age, mean (SD)**	38.3 (13.2)	50.9 (15.7)
**Female gender, *n* (%)**	14,092 (34.8%)	3234 (75.38%)
**Years followed in database, mean (SD) ^1^**	6.4 (3.0)	6.7 (3.3)
**Occupation type, *n* (%)**		
Civil servants, teachers, military personnel, and veterans	2468 (6.06%)	217 (5.06%)
Non-manual workers and professionals	15,795 (38.76%)	1063 (24.78%)
Manual workers	9207 (22.60%)	1514 (35.29%)
Others	3940 (9.67%)	353 (8.23%)
Dependents of insured individuals	7909 (19.41%)	1105 (25.76%)
Missing	1428 (3.50%)	38 (0.89%)
**Urbanization, *n* (%)**		
Urban	25,266 (62.01%)	2589 (60.35%)
Suburban	11,524 (28.28%)	1299 (30.28%)
Rural	2529 (6.21%)	364 (8.48%)
Missing	1428 (3.50%)	38 (0.89%)
**Monthly income level (median USD for 1:30 exchange rate), *n* (%)**		
Quartile 0 (42)	11,569 (28.39%)	1177 (27.44%)
Quartile 1 (670)	9624 (23.62%)	1395 (32.52%)
Quartile 2 (960)	9545 (23.43%)	960 (22.38%)
Quartile 3 (1687)	10,009 (24.56%)	758 (17.67%)
**Inpatient visits within one year prior to the diagnosis, *n* (%)**		
Never	36,598 (89.82%)	3474 (80.98%)
1	3163 (7.76%)	547 (12.75%)
2	697 (1.71%)	150 (3.50%)
≥3	289 (0.71%)	119 (2.77%)
**Elixhauser comorbidity score, *n* (%)**		
0	27,268 (66.92%)	1839 (42.87%)
1	8916 (21.88%)	1218 (28.39%)
2	2988 (7.33%)	644 (15.01%)
3	1023 (2.51%)	330 (7.69%)
≥4	552 (1.35%)	259 (6.04%)
**BASELINE MEDICAL CONTACT**		
**Total outpatient visits during one year prior to index date**		
Mean (SD)	18.95 (15.61)	30.33 (21.49)
Median (IQR)	15 (8–25)	26 (15–40)
**Visits to a rheumatologist in the six months post index date**		
Mean (SD)	2.81 (2.47)	1.01 (2.14)
Median (IQR)	2 (1–4)	0 (0–1)
**Visits to a gastroenterologist in the six months post index date**		
Mean (SD)	0.24 (0.93)	1.00 (2.32)
Median (IQR)	0 (0–0)	0 (0–1)
**PREVENTABLE HOSPITALIZATIONS FOR AMBULATORY CARE SENSITIVE CONDITIONS DURING STUDY PERIOD**		
**Reason for hospitalization, *n* (%)**		
Bacterial Pneumonia	561 (1.38)	273 (6.36)
Urinary Tract Infection Admission Rate	432 (1.06)	243 (5.66)
Diabetes Long-Term Complications	177 (0.43)	88 (2.05)
Diabetes Short-Term Complications	31 (0.08)	20 (0.47)
Heart Failure	77 (0.19)	37 (0.86)
Hypertension	119 (0.29)	72 (1.68)
Uncontrolled Diabetes	55 (0.13)	16 (0.37)
Lower-Extremity Amputation among Patients with Diabetes	0 (0)	1 (0.02)
Overall	1219 (2.89)	551 (12.84)
**Number of total quarters with any preventable hospitalization, *n* (%)**		
1	893 (2.19)	368 (8.58)
2	158 (0.39)	98 (2.28)
3	69 (0.17)	52 (1.21)
4	21 (0.05)	16 (0.37)
5	20 (0.05)	6 (0.14)
≥6	18 (0.04)	11 (0.26)

AS = ankylosing spondylitis; IBD = inflammatory bowel disease. ^1^ Time from enrollment until either death or the end of the study period.

**Table 2:** Corticosteroid usage among patients with ankylosing spondylitis (AS) and inflammatory bowel disease (IBD) during the study period, by calendar quarter.

	Q –1	Q1	Q2	Q3	Q4
**AS patients who received corticosteroids (total *n* = 40,747)**					
*n* (%) Receiving Corticosteroids	4558 (11.2%)	7525 (18.5%)	5346 (13.1%)	4776 (11.7%)	4569 (11.3%)
Median Dose (mg) (IQR)	90.0 (35.0–166.9)	140.0 (56.0–315.0)	105.0 (40.0–320.0)	94.7 (36.0, 280.0)	90.0 (30.0, 280.0)
Mean Dose (SD)	142.0 (217.1)	239.0 (306.3)	237.7 (360.0)	221.6 (362.6)	206.3 (313.1)
Median Duration (days) (IQR)	9.0 (6.0–17.0)	16.0 (6.0–44.0)	14.0 (6.0–56.0)	13.0 (5.0, 53.0)	10.0 (4.0–48.0)
Mean Duration (SD)	14.2 (14.6)	28.4 (26.8)	31.6 (32.7)	29.9 (32.8)	28.2 (31.9)
Median Daily Dose (mg/day) (IQR)	10.0 (4.0, 15.0)	7.9 (4.6, 13.0)	6.6 (3.8, 11.5)	6.7 (3.5, 12.0)	6.7 (3.1, 12.0)
Mean Daily Dose (mg/day) (SD)	10.7 [9.4]	9.7 (8.5)	8.6 (7.7)	8.9 (8.3)	8.9 (8.8)
**IBD patients who received corticosteroids (total *n* = 4290)**					
*n* (%) Receiving Corticosteroids	863 (20.1%)	1298 (30.3%)	1316 (30.7%)	1327 (30.9%)	1289 (30.4%)
Median Dose (mg) (IQR)	120.0 (50.0, 245.6)	280.0 (90.0, 543.0)	280.0 (90.6, 598.9)	308.3 (90.0, 610.0)	324.0 (112.0, 650.0)
Mean Dose (SD)	203.3 (275.8)	387.1 (398.4)	417.7 (450.8)	427.4 (450.1)	444.6 (462.3)
Median Duration (days) (IQR)	16.0 (7.0, 28.0)	39.0 (10.0, 74.0)	42.0 (11.8, 83.0)	50.0 (13.0, 85.0)	51.0 (15.0, 85.0)
Mean Duration (SD)	22.9 (21.3)	43.4 (32.6)	46.5 (34.4)	49.1 (35.3)	50.5 (34.9)
Median Daily Dose (mg/day) (IQR)	7.5 (4.0, 12.0)	8.0 (5.0, 13.7)	7.8 (5.0, 12.5)	7.5 (5.0, 11.9)	7.8 (5.0, 12.0)
Mean Daily Dose (mg/day) (SD)	9.1 (7.5)	9.6 (7.4)	9.4 (7.3)	9.2 (7.2)	9.3 (7.3)

AS = ankylosing spondylitis; IBD = inflammatory bowel disease. ^1^ *n* = 40,360.

**Table 3:** Frequency of corticosteroid and disease modifying anti-rheumatic drug usage among patients with ankylosing spondylitis and inflammatory bowel disease during the study period.

	AS (*n* = 40,747)			IBD (*n* = 4290)		
	User	Non-User	%	User	Non-User	%
**Steroid**	25,928	14,819	63.63%	3677	613	85.71%
**Biologic DMARD**	1210	39,537	2.97%	255	4035	5.94%
Adalimumab	709	40,038	1.74%	134	4156	3.12%
Etanercept	522	40,225	1.28%	134	4156	3.12%
Golimumab	90	40,657	0.22%	10	4280	0.23%
Ustekinumab	4	40,743	0.01%	0	4290	0.00%
**Synthetic DMARD**	27,165	13,582	66.67%	3133	1157	73.03%
Azathioprine	598	40,149	1.47%	602	3688	14.03%
Cyclophosphamide	148	40,599	0.36%	164	4126	3.82%
Cyclosporine	462	40,285	1.13%	262	4028	6.11%
Hydroxychloroquine	3578	37,169	8.78%	2493	1797	58.11%
Leflunomide	607	40,140	1.49%	328	3962	7.65%
Methotrexate	3850	36,897	9.45%	1146	3144	26.71%
Minocycline	3416	37,331	8.38%	355	3935	8.28%
Mycophenolate	30	40,717	0.07%	32	4258	0.75%
Sulfasalazine	24,630	16,117	60.45%	1325	2965	30.89%

AS = ankylosing spondylitis; IBD = inflammatory bowel disease; DMARD = disease-modifying anti-rheumatic drug.

**Table 4:** Adjusted odds ratios of hospitalization by corticosteroid or disease modifying anti-rheumatic drug exposure by calendar quarter.

	Patients with AS (*n* = 40,747)		Patients with IBD (*n* = 4290)	
	Odds Ratio	*p*-Value	Odds Ratio	*p*-Value
**Preventable hospitalization within current quarter**	4.455 (3.672–5.405)	<0.0001	4.376 (3.417–5.605)	<0.0001
**Corticosteroid**	1.055 (1.037–1.074)	<0.0001	1.064 (1.046–1.082)	<0.0001
**Biologic DMARD (CI)**				
Adalimumab	1.289 (0.728–2.283)	0.384	1.725 (0.916–3.250)	0.091
Etanercept	1.790 (1.009–3.177)	0.047	0.649 (0.289–1.457)	0.295
**Synthetic DMARD (CI)**				
Azathioprine	1.273 (0.901–1.799)	0.171	1.106 (0.847–1.444)	0.458
Cyclophosphamide	1.441 (0.802–2.590)	0.221	0.845 (0.495–1.442)	0.537
Cyclosporin	1.002 (0.608–1.650)	0.995	1.078 (0.714–1.627)	0.721
Hydroxychloroquine	1.241 (1.105–1.518)	0.035	1.086 (0.945–1.248)	0.243
Leflunomide	0.850 (0.464–1.557)	0.599	1.042 (0.713–1.523)	0.832
Methotrexate	1.015 (0.964–1.069)	0.573	1.008 (0.810–1.256)	0.940
Minocycline	1.047 (0.848–1.293)	0.670	0.701 (0.313–1.570)	0.387
Sulfasalazine	0.954 (0.873–1.042)	0.294	0.838 (0.675–1.040)	0.108

AS = ankylosing spondylitis; IBD = inflammatory bowel disease; DMARD = disease-modifying anti-rheumatic drug.

## Supplementary Materials

The following are available online at https://www.mdpi.com/2077-0383/8/5/614/s1, Figure S1: Detailed attrition diagram, Figure S2: Timeline of washout period and study period, Figure S3: Method of quantifying overlapping prescriptions, Table S1: Parameters for identifying subjects with AS and IBD, Table S2: Corticosteroids and disease modifying anti-rheumatic drugs assessed in the study, Table S3: Equivalent doses of oral corticosteroids, Table S4: Scaling for disease modifying anti-rheumatic drug medication dosages (in mg), Table S5: Ambulatory care-sensitive conditions likely to be exacerbated by corticosteroid use. As defined by defined by the Agency of Healthcare Quality and Research (AHRQ), Table S6: List of covariates used in primary model, Table S7: Sensitivity analysis showing effect of replacing the standard Elixhauser Comorbidity Index Score with a modified score excluding corticosteroid-sensitive conditions.
