# Supplementary material for: Impact of Cumulative Corticosteroid Dosage on Preventable Hospitalization among Taiwanese Patients with Ankylosing Spondylitis and Inflammatory Bowel Disease
Source: J Clin Med. 2019 May 7;8(5):614. doi: 10.3390/jcm8050614 (PMC6572534; doi:10.3390/jcm8050614)
Supplement: Supplementary file 1 [file jcm-08-00614-s001.pdf]

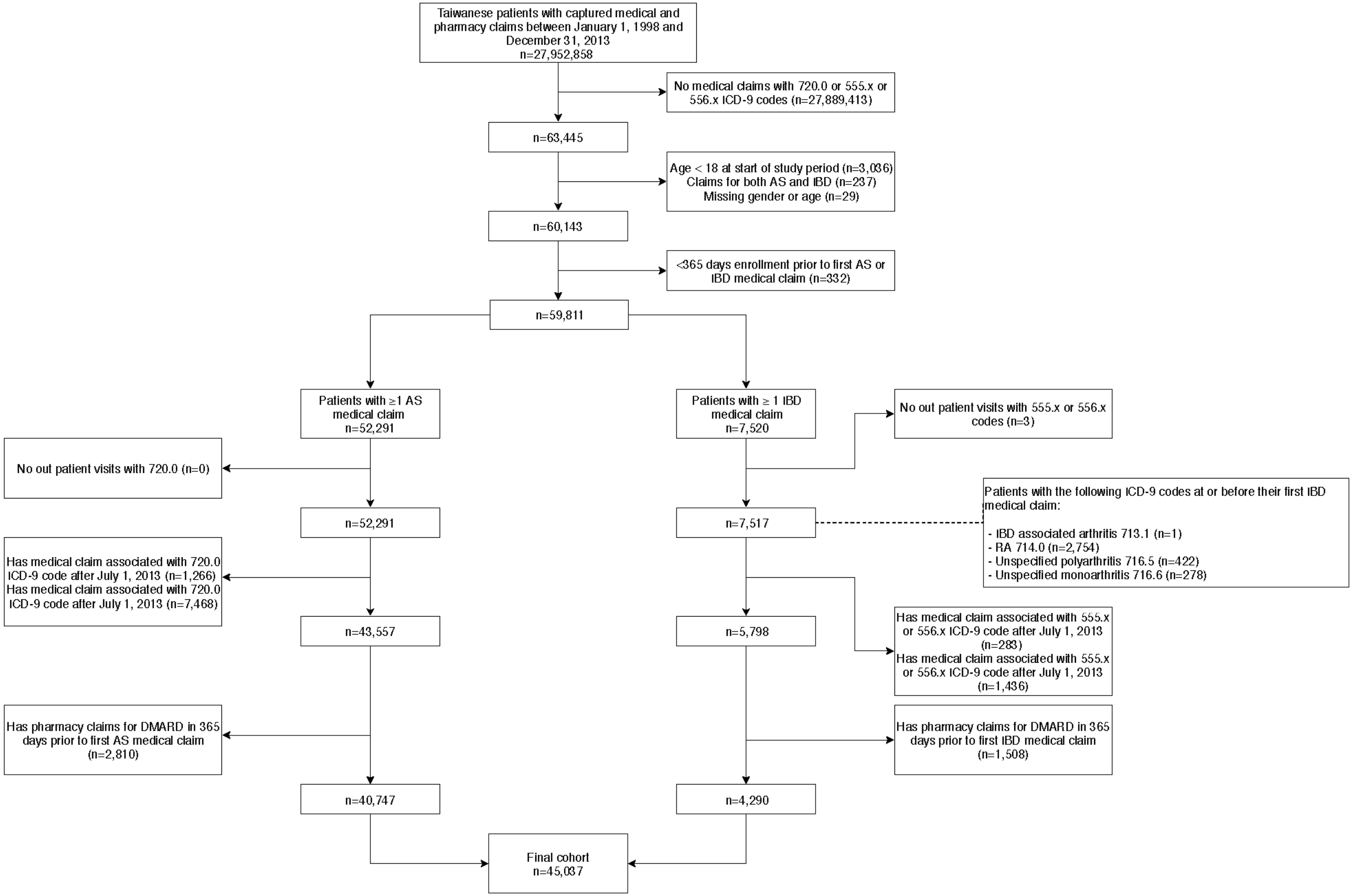

Figure S1. Detailed Attrition Diagram. The study cohort was defined using validated claims definitions identified by Rezaie et al. for inflammatory bowel disease (IBD) and by Singh et al. for ankylosing spondylitis (AS). Patients were excluded if they had any medical claims for AS or IBD or pharmacy claims for disease-modifying anti-rheumatic drugsin the 365 days prior to index diagnosis, had claims for both diseases, were younger than 18 years, or lacked complete demographic data.

Table S1. Parameters for identifying subjects with ankylosing spondylitis and inflammatory bowel disease

Disease	Investigator	Criteria	
**Inflammatory Bowel Disease**	**Rezaie, A et al.**	individuals who experienced at least:<br>• **2 hospitalizations**<br>*or*<br>• **4 physician claims**<br>*or*<br>• **2 medical contacts** in the Ambulatory Care Classification System database<br><br>with an IBD diagnostic code (ICD-9 555.x or 556.x) within a two-year period	• Specificity: 99.8%<br>• Sensitivity: 83.4%<br>• Positive Predictive Value: 97.4%<br>• Negative Predictive Value 98.5%
**Ankylosing Spondylitis**	**Singh, J et al.**	Individuals with >2 ICD-9 codes for AS (720)	• Specificity: 100%<br>• Sensitivity: 82%<br>• Positive Predictive Value: 100%<br>• Negative Predictive Value 99

Table S2. Corticosteroids and disease modifying anti-rheumatic drugs assessed in the study.

Class	Medications
Synthetic DMARD	Azathioprine, Cyclophosphamide, Cyclosporin, Hydroxychloroquine, Leflunomide, Methotrexate, Minocycline, Mycophenolate, Sulfasalazine
Biologic DMARD and Janus Kinase inhibitors	Abatacept, Adalimumab, Etanercept, Golimumab, Rituximab, Tocilizumab, Tofacitinib, Ustekinumab

Table S3. Equivalent doses of oral corticosteroids.

Glucocorticoid	Dose	Glucocorticoid	Dose
Betamethasone	0.6 mg	Prednisone	5 mg
Dexamethasone	0.75 mg	Prednisolone	5 mg
Methylprednisolone	4 mg	Hydrocortisone	20 mg
Triamcinolone	4 mg	Cortisone	25 mg

Table S4: Scaling for disease modifying anti-rheumatic drug medication dosages (in mg)

	**Median cumulative dose per three-month calendar quarter (mg)**	
	AS Group	*IBD Group*
**Adalimumab**	220	209
**Etanercept**	496	543
**Azathioprine**	2800	3750
**Cyclophosphamide**	2000	1400
**Cyclosporin**	5250	5200
**Hydroxychloroquine**	16,600	18,200
**Leflunomide**	980	1020
**Methotrexate**	80	99
**Minocycline**	800	900
**Sulfasalazine**	56,000	84,000

Table S5. Ambulatory care-sensitive conditions likely to be exacerbated by corticosteroid use**.** As defined by the Agency of Healthcare Quality and Research (AHRQ).

**Condition**	**Code**	**ICD-9-CM[1] disease code**	**Excluded criteria[2]**
Uncontrolled diabetes	Primary dx	250.02, 250.03	
Diabetes, short-term complications	Primary dx	250.10-250.13, 250.20-250.23, 250.30-250.33	
Diabetes, long-term complications	Primary dx	250.40-250.43, 250.50-250.53, 250.60-250.63, 250.70-250.73, 250.80-250.83, 250.90-250.93	
Lower extremity amputation among patients with diabetes	In any field	250 and procedure code of 841.0, 841.2-841.9	Excludes any-listed diagnosis of traumatic lower-extremity amputation admissions and obstetric admissions.
Hypertension	Primary dx	401.0, 401.9, 402.00, 402.10, 402.90, 403.00, 403.10, 403.90, 404.00, 404.10, 404.90	Excludes kidney disease combined with dialysis access procedure admissions, cardiac procedure admissions, and obstetric admissions,
Congestive heart failure	Primary dx	398.91, 402.01, 402.11, 402.91, 404.01, 404.03, 404.11, 404.13, 404.91, 404.93, 428.0-428.1, 428.20-428.23, 428.30-428.33, 428.40-428.43, 428.9	Excludes cardiac procedure admissions and obstetric admissions.
Bacterial pneumonia	Primary dx	481, 485, 486, 482.2, 482.9, 483.0-483.1, 483.8, 482.30-482.32, 482.39-482.42, 482.49	Excludes sickle cell or hemoglobin-S admissions, other indications of immunocompromised state admissions, and obstetric admissions.
Urinary tract infection	Primary dx	590.10-590.11, 590.2-590.3, 590.80-590.81, 590.9, 595.0, 595.9, 599.0	Excludes kidney or urinary tract disorder admissions, other

indications of immunocompromised state admissions, and obstetric admissions.

[1]International Classification of Diseases, 9th revision, Clinical Modification
[2]For the excluded criteria, refer to the website of AHRQ (https://www.qualityindicators.ahrq.gov/modules/pqi_resources.aspx) to find the detailed ICD-9-CM code.

Table S6: List of covariates used in primary model.

**Type**	**Variable Names**
Demographic Variables	• Age<br>• Gender<br>• Occupation type<br>• Urbanization<br>• Income level
Health Condition Variables	• Elixhauser comorbidity score<br>• Inpatient visits within one year prior to index date<br>• Total outpatient visits during one year prior to index date<br>• Visits to a rheumatologist in the six months post index date (AS only)<br>• Visits to a gastroenterologist in the six months post index date (IBD only)
Main variables	• Having any preventable hospitalization within current quarter<br>• Steroid<br>• Biological DMARD<br>o Adalimumab<br>o Etanercept<br>• Synthetic DMARD<br>o Azathioprine<br>o Cyclophosphamide<br>o Cyclosporin<br>o Hydroxychloroquine<br>o Leflunomide<br>o Methotrexate<br>o Minocycline<br>o Sulfasalazine

Table S7: Sensitivity analysis showing effect of replacing the standard Elixhauser comorbidity index score with a modified score excluding corticosteroid-sensitive conditions

**Variable Name**	**Patients with AS (*n* = 40747)**		**Patients with IBD**	
	**Odds Ratio**	***p*-Value**	**Odds Ratio**	***p*-value**
**Having any preventable hospitalization within current quarter**	4.656 [3.836-5.652]	<0.0001	4.360 [3.403-5.585]	<0.0001

	Corticosteroid effect on hospitalization among Taiwanese rheumatologic patients			
**Steroid**	1.054 [1.036-1.072]	<0.0001	1.064 [1.047-1.082]	<0.0001
**Biologic DMARD [CI]**				
Adalimumab	1.335 [0.756-2.356]	0.319	1.714 [0.909-3.229]	0.096
Etanercept	1.728 [0.965-3.095]	0.066	0.662 [0.294-1.490]	0.319
**Synthetic DMARD (CI)**				
Azathioprine	1.282 [0.908-1.809]	0.158	1.112 [0.852-1.453]	0.434
Cyclophosphamide	1.438 [0.806-2.565]	0.219	0.850 [0.497-1.453]	0.553
Cyclosporin	0.986 [0.600-1.619]	0.955	1.074 [0.711-1.620]	0.736
Hydroxychloroquine	1.244 [1.018-1.520]	0.033	1.087 [0.946-1.249]	0.237
Leflunomide	0.862 [0.474-1.568]	0.627	1.038 [0.711-1.515]	0.846
Methotrexate	1.012 [0.960-1.067]	0.663	1.008 [0.810-1.255]	0.941
Minocycline	1.043 [0.844-1.288]	0.698	0.701 [0.312-1.572]	0.388
Sulfasalazine	0.974 [0.892-1.062]	0.549	0.838 [0.675-1.040]	0.109

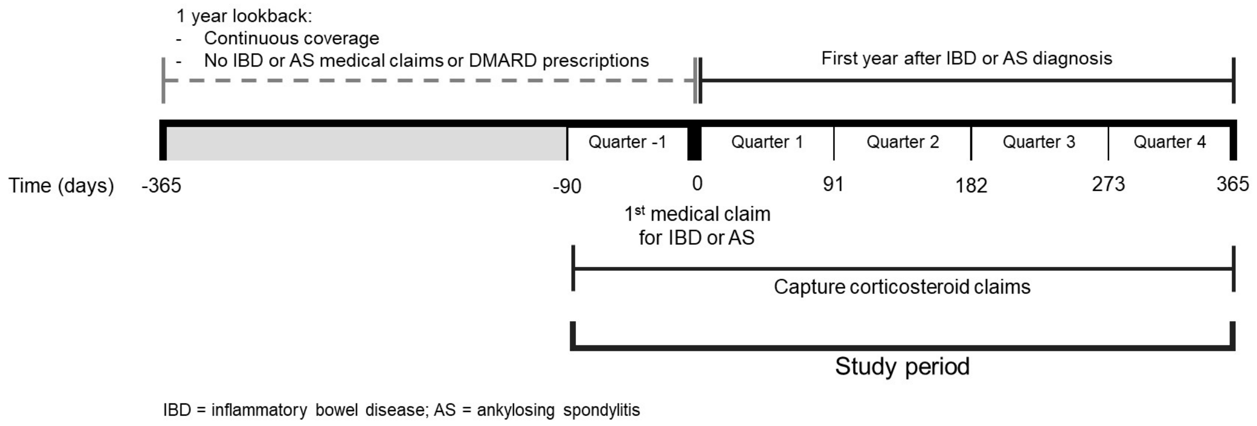

Figure S2. Timeline of washout period and study period.

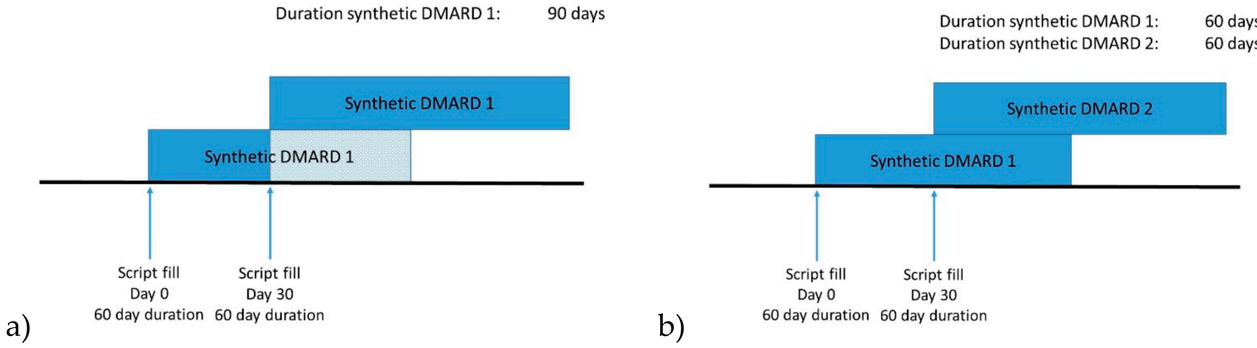

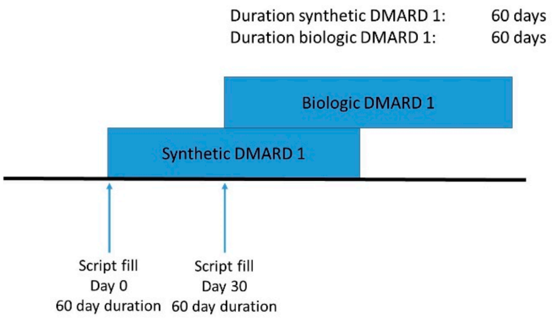

c)

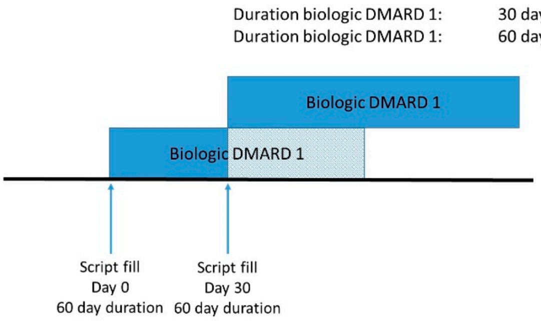

d)

Figure S3. Method of quantifying overlapping prescriptions for (**a**) the same synthetic DMARD, (**b**) two different synthetic DMARDs, (**c**) a synthetic DMARD and a biologic, and (**d**) two biologics.
